# Apathy Is Associated with Slower Gait and Subjective Cognitive Complaints in a South Indian Community-Dwelling Cohort

**DOI:** 10.3390/brainsci15111204

**Published:** 2025-11-07

**Authors:** Matthew G. Engel, Emmeline I. Ayers, Dristi Adhikari, Marnina B. Stimmel, Erica F. Weiss, V.G. Pradeep Kumar, Alben Sigamani, Joe Verghese, Mirnova E. Ceïde

**Affiliations:** 1 Psychiatry Residency Program, Johns Hopkins Hospital, Baltimore, MD 21287, USA; mengel11@jh.edu; 2 Department of Neurology, Renaissance School of Medicine, Stony Brook, NY 11794, USA; emmeline.ayers@stonybrookmedicine.edu (E.I.A.); dristi.adhikari@stonybrookmedicine.edu (D.A.);; 3 Department of Neurology, Albert Einstein College of Medicine, Bronx, NY 10461, USA; marstimm@montefiore.org (M.B.S.); eweiss@montefiore.org (E.F.W.); 4 Ba’by Memorial Hospital, Kozhikode 673004, Kerala, India; vgpradeep@hotmail.com; 5 Numen Health, Bangalore 560095, Karnataka, India; dralbens@myrescon.com; 6 Department of Psychiatry and Behavioral Sciences and Medicine, Albert Einstein College of Medicine, Bronx, NY 10461, USA

**Keywords:** apathy, MCR, dementia, gait, aging

## Abstract

**Background/Objectives**: Apathy is an independent risk factor for dementia and motoric–cognitive risk syndrome (MCR), a predementia syndrome characterized by slow gait and subjective cognitive complaints (SCCs). Our objective is to assess the cross-sectional association of apathy with gait velocity, SCC, and MCR in a community-based cohort of older adults. **Methods**: A cross-sectional survey of N = 746 community-dwelling older adults (≥60 years of age) enrolled in the Kerala Einstein Study. Apathy was measured using the Apathy Evaluation Scale (AES). Participants were stratified by AES tertile to evaluate bivariate associations, and multivariate linear and logistic regression models were used to assess the relationship of apathy with gait velocity, SCC, and MCR. **Results**: Compared with participants in the lowest apathy tertile, those in the highest tertile were significantly older, less physically active, and had slower gait. High-apathy participants also had lower Addenbrooke’s Cognitive Examination scores (79.4 vs. 84.5, *p* < 0.001) and higher depression scores (9.3 vs. 5.4, *p* < 0.001). Apathy was associated with slower gait velocity (β = −3.465, *p* ≤ 0.002), but this relationship was no longer significant after adjusting for ACE score. Apathy and SCC were significantly associated in adjusted models (*p* < 0.001). Although participants with MCR had higher levels of apathy compared to those without MCR (34.6 vs. 31.4, *p* < 0.01), prevalent MCR and apathy were not significantly associated in regression models. **Conclusions**: Among community-dwelling older adults in Kerala, apathy is associated with slower gait and more severe subjective cognitive complaints but not cross-sectional MCR prevalence. These findings suggest that apathy may serve as an early risk factor in dementia pathogenesis across diverse patient populations, warranting further longitudinal investigation.

## 1. Introduction

Apathy is a clinical syndrome characterized by a primary loss of motivation not due to reduced consciousness, emotional disturbance, or intellectual impairment [1,2]. Apathy has been widely shown to precede the development of age-related cognitive decline and motor impairment [3,4]. Prodromal apathy is seen in Parkinson’s disease (PD) and Lewy body dementia [5,6], Alzheimer’s disease (AD) [7], frontotemporal dementia (FTD) [8], Huntington disease (HD) [9], and multiple sclerosis (MS) [10], suggesting a shared pathway by which early inflammatory and neurodegenerative processes promote an apathy state before progression to clinically significant disease. Our group has previously identified an association between apathy and incident motoric cognitive risk syndrome (MCR), a predementia syndrome defined by the combination of subjective cognitive complaints (SCCs) plus slow gait velocity [11].

SCCs, a component of MCR, are frequently seen in older adults and often precede the onset of dementia [12]. Stratification of older adults with subjective cognitive impairment according to risk level is a major public health priority, as early lifestyle and clinical interventions may stave off cognitive decline in higher-risk patients [13]. Slow gait is also a risk factor for age-related cognitive decline and is especially predictive of non-Alzheimer’s disease risk [14]. In the United States, the incidence of MCR in older adults has been estimated at 65.2 per 1000 person-years, with increased risk observed with concomitant depressive symptoms, obesity, and sedentary lifestyle [15].

The relationships between apathy, MCR, and dementia have been studied extensively in mostly non-Hispanic White community cohorts within high-income countries. With rapid demographic shift owing to reduced infant mortality and substantially increased life expectancy, the population of older adults in India is expected to continue to grow at a rate of about 3.6 percent per year [16]. Thus, identifying non-invasive screening instruments, such as the Apathy Evaluation Scale (AES), to risk-stratify older Indian adults is increasingly important in the community setting. Our objective was to assess the cross-sectional association between apathy and motor–cognitive outcomes, such as gait velocity, SCC, and MCR, in a community-dwelling cohort of older adults residing in the south Indian state of Kerala.

## 2. Materials and Methods

N = 746 participants enrolled in the Kerala Einstein Study were included in this study. This was a cross-sectional study of older adults (≥60 years old) in urban and rural locations in Kozhikode district, a northern district in Kerala state with a population of more than 2 million people. Participants recruited from the urban site attended the neurology clinic at Baby Memorial Hospital (BMH), while rural and exurban recruitment sites included a BMH community health center in Kakkodi village and Meitra Hospital-affiliated outpatient clinics, respectively. Inclusion criteria were (1) age > 60 years and (2) ability to provide informed consent. Exclusion criteria were (1) refusal to complete study tasks (completion rate > 95% in initial KES phase), (2) previous diagnosis of dementia, (3) acute or terminal illness, or (4) severe auditory or visual impairment [17]. The aims of the study were to examine the association of apathy, a potentially modifiable risk factor, and MCR, a predementia syndrome. This study was conducted in accordance with the Helsinki Declaration. The informed consent and recruitment processes follow local socially and culturally appropriate practices. The assessments and procedures have been approved by the Ethics Board at Baby Memorial Hospital. The study design and consent procedures were approved by the Indian Council of Medical Research (ICMR). The study protocols have also been reviewed and approved by the Albert Einstein College of Medicine IRB.

### 2.1. Apathy

Apathy was measured using the Apathy Evaluation Scale (AES), an 18-item questionnaire that assesses apathy symptom severity in three domains (behavioral, emotional, and cognitive) as the criterion standard [1]. The AES has been validated in numerous languages, including Malayalam, and exhibits excellent test–retest and interrater reliability (as described by Clarke et al., α = 0.76–0.94 at 25.4 days; intraclass correlation coefficient = 0.94) [18,19]. For tertile analyses, the total cohort was split into three nearly equivalently sized sub-groups based on AES score (Tertile 1/Low Apathy ≤ 25 (n = 222), Tertile 2/Medium Apathy = 26–35 (n = 225), Tertile 3/High Apathy ≥ 36 (n = 226)).

### 2.2. GDS

Depression was assessed using the 30-item Geriatric Depression Scale (GDS) [20]. The strength of the GDS in older adult populations is that it was developed for use in older adults, including those with somatic and cognitive complaints. GDS has been shown to be a reliable screening tool in people with mild cognitive impairment across numerous populations.

### 2.3. MCR

MCR was operationalized as the presence of two or more cognitive complaints and slow gait in participants without dementia or mobility disability (inability to ambulate even with assistance or walking aids). Subjective cognitive complaints were defined by responses to memory-related questions in the Cognitive Change Index (CCI), Kerala Activities of Daily Living Questionnaire (ADL), and Geriatric Depression Scale (GDS). Gait was assessed with a 3.5 m timed walk on a straight computerized mat with embedded pressure sensors (GAITRite^®^, CIR Systems, Inc., Franklin, NJ, USA). Study participants walked at a typical pace indoors with adequate lighting and without distractions. Slow gait was defined as 1 SD below age- and sex-adjusted means among Indian adults (<59.6 cm/s for men and <52.6 cm/s for women under 75 years, and <44.7 cm/s for men and <37.5 cm/s for women age ≥ 75 years) [21]. MCR was diagnosed if all the following criteria were met: (1) subjective cognitive complaints, as defined above; (2) slow gait; and (3) absence of dementia (picture-based memory impairment screen (PMIS) ≤ 5 [22].

### 2.4. Subjective Cognitive Change (SCC)

Subjective cognitive change (SCC) was operationalized as a continuous variable derived from the Cognitive Change Index (CCI), a twenty-item questionnaire broadly used for the assessment of perceived cognitive decline [23]. The self-rating scale asked participants about their perceived cognitive abilities, daily functioning, and activities, compared with 5 years ago, with a Likert score of 1–5 selected for each item (1 = normal ability, 2 = slight/occasional problem, 3 = mild problem, 4 = moderate problem, 5 = severe problem). Higher sums on the CCI-20 correspond with more severe SCC.

### 2.5. Covariates

Age was self-reported by study participants. Dysphoria was calculated as an eight-item sub-score from the GDS based on factor analysis, as previously described [24]. Addenbrooke’s Cognitive Examination-III (ACE) comprises a global cognition tool assessing memory, language, visuospatial performance, orientation, and attention, administered in participants’ preferred language (Malayalam or English). Cutoffs have been defined to detect cognitive impairment; here, the ACE score was treated as a continuous variable [25]. General health score reflects a subjective self-assessment (1 = Poor, 5 = Excellent) in response to the prompt, “In general, would you say your health is…” Physically active days were treated as a continuous variable, representing the mean number of days per week for which physical activity was reported.

### 2.6. Statistical Methods

Statistical analysis was completed using SPSS version 29.0 (SPSS Inc., Chicago, IL, USA). Bivariate analyses were conducted of the baseline characteristics of the participants. Independent Student’s *t*-test was used for continuous variables, and Chi-square tests were used to evaluate categorical variables based on MCR status. ANOVA was used to evaluate bivariate associations by apathy tertile status. Covariates, including age, dysphoria, Addenbrooke’s Cognitive Examination (ACE) score, general health score, and physical activity days, were added stepwise to regression models based on the significance of the associations in the bivariate analyses. Linear regression models were built to evaluate the association between apathy (AES tertile), gait velocity, and subjective cognitive complaints (based on CCI). Logistic regression models were utilized to assess the association between apathy (AES tertile) and MCR (see Supplementary Materials).

## 3. Results

### 3.1. Baseline Characteristics

A total of 673 participants were included in the study (initial N = 746, with n = 73 excluded due to dementia based on PMIS ≤ 5) (Table 1). Compared with MCR-free participants, those with MCR had fewer years of education, were less likely to be working, and scored higher on AES (34.6 vs. 31.4, *p* ≤ 0.01). MCR participants also performed worse on ACE, a measure of cognitive performance (77.0 vs. 82.4, *p* ≤ 0.005), reported greater levels of depression assessed by the GDS (9.2 vs. 6.7, *p* ≤ 0.005) but not dysphoria per se, and reported more severe SCC based on CCI score (41.4 vs. 33.8, *p* ≤ 0.001).

Demographics according to the apathy tertile are shown in Table 2. Compared with those in the lowest tertile, participants in the highest apathy tertile were older, less physically active, reported worse general health, and exhibited slower gait (74.7 vs. 82.5 cm/s, *p* ≤ 0.001). Additionally, participants in the highest apathy tertile performed worse on ACE (79.4 vs. 84.5, *p* ≤ 0.001). Scores on GDS were higher in the highest apathy tertile (9.3 vs. 5.4, *p* ≤ 0.001), but no difference was observed in GDS dysphoria sub-scores among groups. SCC increased in a stepwise manner with higher levels of apathy (*p* ≤ 0.001).

### 3.2. Regression Models

Higher apathy tertile correlated with slower gait (Model 1, β = −3.465, *p* = 0.002) (Table 3). This relationship persisted after adjusting for age and dysphoria. However, the relationship was no longer significant after adjusting for ACE score and was further attenuated after accounting for self-reported general health and physical activity frequency (Model 6, adjusted β = −1.569, *p* = 0.137). Apathy and SCC showed a positive association that remained significant after adjustment for age, dysphoria, ACE score, self-reported general health, and physically active days per week (Table 4; Model 6, adjusted β = +4.666, *p* ≤ 0.001).

Apathy tertiles and prevalent MCR were positively associated, but this relationship did not reach significance (Table S1; OR = 1.83, *p* = 0.078). No relationship was seen between dysphoria and MCR (Table S2; OR = 0.94, *p* = 0.870). However, a positive relationship between GDS and MCR without adjustment (Model 1, OR = 2.62, *p* = 0.005) that weakened after adjusting for self-reported general health (Model 4, OR = 1.58, *p* = 0.232) (Table S3).

## 4. Discussion

In this cross-sectional study of community-dwelling older adults residing in the south Indian state of Kerala, apathy was associated with slower gait and increased SCC but not prevalent MCR. The relationship between apathy and gait velocity persisted after adjustment for age and dysphoria but was moderated by ACE score, subjective health status, and physical activity, whereas the association between apathy and SCC remained highly significant even after adjustment.

The interrelation of apathy, gait slowing, and SCC reported here reflects the well-established triad of apathy, slow gait, and executive dysfunction (AGED) shown previously to predict dementia risk [26]. Although beyond the scope of this study, cerebrovascular disease may underlie the AGED triad, especially in older adults with multiple vascular risk factors, such as type 2 diabetes, hypertension, obesity, and physical inactivity [26,27,28]. On the other hand, apathy is an independent risk factor for subjective cognitive changes even after adjusting for comorbid chronic disease in older adults, suggesting the presence of additional mediators between apathy and self-reported cognitive decline, such as systemic inflammation, as we and others have previously demonstrated [12,29,30]. The neurobiology of apathy is thought to involve dysfunctional frontostriatal circuitry, leading to diminished executive function and motivational behaviors [31]. For example, shared involvement of the dorsolateral prefrontal cortical circuit in both apathy and cognitive complaints may partially explain their correlation in cross-section, although analysis of circuit dysfunction is beyond the scope of this study and warrants further investigation in the KES cohort [32,33].

Interestingly, in contrast with previous work revealing a significant association between apathy and incident MCR in the Central Control of Mobility in Aging (CCMA) cohort, in this study, we found that apathy was associated only with constituent motoric cognitive outcomes (i.e., gait velocity and SCC) as independent variables rather than MCR per se [11]. Notably, the magnitudes of association between apathy and gait velocity as well as apathy and MCR were attenuated after adjustment for ACE score, an objective measure of cognitive function. In the context of a strong association between apathy and SCC even after adjustment for ACE score, as well as prior evidence that SCC can precede clinically significant cognitive decline, it is plausible that the present study captures an earlier cross-section of a common neuropathologic continuum in the Kerala Aging cohort, which was also noted in the CCMA study population, in which apathy was associated with slower gait but not MCR in a cross-sectional analysis [29,34]. Similarity of questions in the AES and CCI instruments may also contribute to the strength of the association between apathy and SCC. We did not perform a direct comparative analysis of these two cohorts but note that they differ with respect to median age and sex ratio, with those in the Kerala study being almost eight years younger (68.6 versus 76.0 years) and with fewer female participants (40.6% versus 55.2%) [11]. Given the association between both age and female sex with late-onset cognitive decline, this demographic disparity may offer some insight into the comparatively modest relationship between apathy and MCR observed here [35,36]. Nevertheless, in both the Kerala and CCMA cohorts, we have observed a significant association between apathy and SCC [24]. It is plausible that cultural factors, such as stigma toward mental health and varying degrees of family support, influence self-reported apathy or SCC. For example, in collectivist cultures such as India, the burden of care for older persons experiencing cognitive decline may preferentially fall on family members instead of healthcare providers, and delays in diagnosis and identification of pre-dementia symptoms (including apathy and SCC) may occur due to patients and their families seeking spiritual instead of medical support [37].

In this study, we found that apathy, but not dysphoria, was associated with cognitive complaints. These findings corroborate previous studies distinguishing apathy from dysphoria, suggesting that apathy is a distinct risk factor for motoric–cognitive outcomes [38,39,40]. Apathy items from the Hamilton Depression Rating Scale, for example, correlate with greater functional impairment in dementia-free older adults with depression, independent of depression severity [41]. Moreover, older adults with concomitant MCI and apathy have been shown to be at heightened risk for progression to dementia compared with those with MCI and depression and those with MCI alone [39]. A longitudinal study of US older adults found that older adults with multimorbid MCI, apathy, and depression were at the greatest risk for developing AD [42]. Although distinct neuropsychiatric disturbances are highly prevalent and often comorbid in older adults with MCI or dementia, a large and growing body of evidence identifies apathy and depression as unique modifiers of cognitive risk, reflecting distinct neuroanatomic and pathologic origins [38,43]. This work thus builds on previous work suggesting that apathy is an important predictor of predementia syndromes and likely occurs early in dementia pathogenesis.

This study has several key limitations. Cross-sectional study design precludes assessment of temporal relationships between apathy, gait velocity, and MCR in the Kerala cohort. For example, we cannot rule out that slow gait and sedentary lifestyle promote apathy without impacting MCR risk. Although we adjusted for several key variables, there may be other competing risk factors, such as previous TBI or stroke, which themselves have strong relationships with apathy, thereby attenuating the association between apathy and MCR in this study. Further, as with all self-reported data, subjective assessments of physical activity and general health imprecisely correlate with objective measures. Additionally, recall bias for self-reported physical activity and health ratings may affect observed associations with apathy, especially in those with concomitant cognitive deficits. However, in this study, self-reported general health was found to vary with MCR status and apathy tertile and therefore constituted a useful correlate of motoric–cognitive function.

## 5. Conclusions

Among community-based older adults in Kerala, apathy is associated with slower gait and more severe SCC but not prevalence of MCR. We are among the first to investigate the relationship between apathy and motoric–cognitive outcomes in an Indian community-dwelling cohort. This study contributes to a growing body of evidence identifying apathy as a distinct cognitive risk factor and may potentially inform the development of screening strategies and Kerala-based public health initiatives in the ambulatory setting.

## Figures and Tables

**Table 1:** Comparison of participants according to MCR status.

Variables	No MCR	MCR	*p*-Value
	N = 600	N = 73	
**Gender, female, % (N)**	39.9% (258)	42.9% (33)	0.614
**Age, mean (SD), years**	68.4 (5.7)	69.5 (5.6)	0.123
**Level of education, mean (SD), years**	9.3 (3.4)	7.4 (3.4)	≤0.001 *
**Currently working (%)**	20.2%	10.4%	0.038 *
**Married (%)**	86.6%	89.6%	0.453
**ACE score, mean (SD)**	82.4 (9.6)	77.0 (12.0)	0.001 *
**Apathy score, mean (SD)**	31.4 (9.9)	34.6 (11.1)	0.010 *
**GDS score, mean (SD)**	6.7 (5.6)	9.2 (6.2)	≤0.001 *
**GDS dysphoria sub-score, mean (SD)**	2.4 (1.4)	2.4 (1.5)	0.877
**General health, mean (SD)**	2.4 (0.7)	2.1 (0.6)	≤0.001 *
**Physically active days per week, mean (SD)**	1.1 (2.5)	1.2 (2.6)	0.875
**Gait velocity, mean (SD), cm/s**	82.5 (18.9)	43.9 (8.4)	≤0.001 *
**CCI sum, mean (SD)**	33.8 (12.2)	41.4 (16.4)	≤0.001 *

* Statistically significant *p*-value < 0.05.

**Table 2:** Baseline demographics by apathy tertiles.

Variables	Total Sample	Low Apathy (AES ≤ 25)	Medium Apathy (AES 26–35)	High Apathy (AES ≥ 36)	*p*-Value
	N = 673	N = 222	N = 225	N = 226	
**Gender, female, % (N)**	38.6% (260)	42.8% (95)	38.7% (87)	34.5% (78)	0.198
**Age, mean (SD), years**	68.5 (5.6)	67.4 (5.5)	68.3 (5.3)	69.6 (5.7)	≤0.001 *
**Level of education, mean (SD), years**	9.2 (3.4)	9.2 (3.3)	9.2 (3.3)	9.1 (3.7)	0.965
**Currently working (%)**	19.8%	23.9%	16.9%	18.6%	0.154
**Married (%)**	88.9%	86.5%	89.3%	90.1%	0.421
**ACE score, mean (SD)**	81.9 (9.9)	84.5 (8.6)	81.7 (9.2)	79.4 (11.4)	≤0.001 *
**Apathy Evaluation Score, mean (SD)**	31.6 (10.0)	21.7 (2.1)	29.4 (2.6)	43.5 (6.5)	≤0.001 *
**GDS score, mean (SD)**	6.9 (5.7)	5.4 (5.2)	5.9 (5.1)	9.3 (5.9)	≤0.001 *
**GDS dysphoria sub-score, mean (SD)**	2.3 (1.4)	2.4 (1.3)	2.2 (1.3)	2.4 (1.5)	0.499
**General health, mean (SD)**	2.4 (0.7)	2.5 (0.7)	2.4 (0.7)	2.3 (0.7)	0.030 *
**Physically active days per week, mean (SD)**	1.2 (2.5)	1.5 (2.8)	1.1 (2.4)	0.9 (2.2)	0.030 *
**Gait velocity, mean (SD), cm/s**	78.8 (22.0)	82.5 (23.3)	79.2 (21.2)	74.7 (20.9)	≤0.001 *
**CCI sum, mean (SD)**	34.6 (12.9)	29.4 (8.0)	32.5 (11.9)	41.9 (14.4)	≤0.001 *
**MCR (%)**	10.8%	8.1%	10.2%	14.2%	0.112

* Statistically significant *p*-value < 0.05.

**Table 3:** Linear regression of apathy and gait velocity.

Independent Variable	Gait Velocity	
Model	Beta Coefficient (95% CI LB, UB)	*p*-Value
Model 1	−3.465 (−5.601, −1.330)	0.002 *
Model 2	−3.041 (−5.186, −0.897)	0.006 *
Model 3	−3.002 (−5.148, −0.856)	0.006 *
Model 4	−1.866 (−3.988, +0.255)	0.085
Model 5	−1.665 (−3.729, +0.399)	0.114
Model 6	−1.569 (−3.637, +0.500)	0.137

* Statistically significant *p*-value ≤ 0.05. Model 1: Apathy tertile. Model 2: +Age. Model 3: +Dysphoria sub-score. Model 4: +ACE score. Model 5: +General health. Model 6: +Physically active days per week.

**Table 4:** Linear regression of apathy and subjective cognitive changes.

Independent Variable	CCI	
Model	Beta Coefficient (95% CI LB, UB)	*p*-Value
Model 1	+5.432 (+4.307, +6.557)	≤0.001 *
Model 2	+5.282 (+4.148, +6.416)	≤0.001 *
Model 3	+5.246 (+4.113, +6.379)	≤0.001 *
Model 4	+4.791 (+3.657, +5.924)	≤0.001 *
Model 5	+4.706 (+3.591, +5.822)	≤0.001 *
Model 6	+4.666 (+3.548, +5.785)	≤0.001 *

* Statistically significant *p*-value ≤ 0.05. Model 1: Apathy tertile. Model 2: +Age. Model 3: +GDS, Dysphoria sub-score. Model 4: +ACE score. Model 5: +General health. Model 6: +Physically active days per week.

## Data Availability

The data are not publicly available due to privacy concerns. However, data are available from the corresponding author upon reasonable request.

## Supplementary Materials

The following supporting information can be downloaded at: https://www.mdpi.com/article/10.3390/brainsci15111204/s1, Table S1: Logistic Regression of Apathy and MCR; Table S2: Logistic regression of dysphoria and MCR; Table S3: Logistic regression of GDS score and MCR; Table S4: Linear regression of GDS score and gait velocity; Table S5: Linear regression of dysphoria and gait velocity; Table S6: Linear regression of GDS score and subjective cognitive changes; Table S7: Linear regression of dysphoria and subjective cognitive changes.

## Abbreviations

The following abbreviations are used in this manuscript:

MCR	Motoric–Cognitive Risk Syndrome
SCCs	Subjective Cognitive Complaints
AES	Apathy Evaluation Scale
CCI	Self-Perceived Cognitive Decline
GDS	Geriatric Depression Scale
