# Supplementary material for: Apathy Is Associated with Slower Gait and Subjective Cognitive Complaints in a South Indian Community-Dwelling Cohort
Source: Brain Sci. 2025 Nov 7;15(11):1204. doi: 10.3390/brainsci15111204 (PMC12651322; doi:10.3390/brainsci15111204)
Supplement: Supplementary file 1 [file brainsci-15-01204-s001.zip › brainsci-3913181-supplementary.pdf]

**Table S1: Logistic Regression of Apathy and MCR**		
**Independent Variable**	Prevalent MCR	
**Model**	OR (95% CI)	p-value
**Model 1**	1.83 (0.94 – 3.59)	0.078
**Model 2**	1.81 (0.92 – 3.56)	0.088
**Model 3**	1.80 (0.91 – 3.54)	0.092
**Model 4**	1.47 (0.73 – 2.96)	0.280
**Model 5**	1.44 (0.71 – 2.91)	0.313
**Model 6**	1.44 (0.71 – 2.92)	0.311

*** Statistically significant p-value ≤ 0.05**
Model 1: Apathy tertile. Model 2: +Age. Model 3: +GDS, Dysphoria sub-score. Model 4: +ACE score. Model 5: +General health. Model 6: +Physically active days per week

**Table S2: Logistic Regression of Dysphoria and MCR**		
**Independent Variable**	Prevalent MCR	
**Model**	OR (95% CI)	p-value
**Model 1**	0.94 (0.46 – 1.94)	0.870
**Model 2**	0.95 (0.46 – 1.97)	0.897
**Model 3**	0.93 (0.45 – 1.91)	0.833
**Model 4**	0.97 (0.47 – 2.04)	0.943
**Model 5**	1.06 (0.51 – 2.23)	0.877
**Model 6**	1.06 (0.50 – 2.23)	0.878

*** Statistically significant p-value ≤ 0.05**
Model 1: GDS, Dysphoria tertile. Model 2: +Age. Model 3: +Apathy score. Model 4: +ACE score. Model 5: +General health. Model 6: +Physically active days per week

**Table S3: Logistic Regression of GDS Score and MCR**		
**Independent Variable**	Prevalent MCR	
**Model**	OR (95% CI)	p-value
**Model 1**	2.62 (1.34 – 5.11)	0.005 *
**Model 2**	2.65 (1.36 – 5.18)	0.004 *
**Model 3**	2.09 (1.04 – 4.20)	0.039 *
**Model 4**	1.58 (0.75 – 3.32)	0.232
**Model 5**	1.58 (0.75 – 3.34)	0.227

*** Statistically significant p-value ≤ 0.05**
Model 1: GDS tertile. Model 2: +Age. Model 3: +ACE score. Model 4: +General health. Model 5: +Physically active days per week

**Table S4: Linear Regression of GDS Score and Gait Velocity**		
**Independent Variable**	Gait Velocity	
**Model**	Beta Coefficient (95% CI LB, UB)	p-value
**Model 1**	-4.545 (-6.680. -2.411)	≤0.001 *
**Model 2**	-4.650 (-6.767, -2.533)	≤0.001 *
**Model 3**	-3.167 (-5.304, -1.031)	0.004 *
**Model 4**	-1.069 (-3.301, +1.163)	0.347
**Model 5**	-0.948 (-3.187, +1.291)	0.406
*** Statistically significant p-value ≤ 0.05**		
**Model 1: GDS tertile. Model 2: +Age. Model 3: +ACE score. Model 4: +General health. Model 5: +Physically active days per week**		

**Table S5: Linear Regression of Dysphoria and Gait Velocity**		
**Independent Variable**	Gait Velocity	
**Model**	Beta Coefficient (95% CI LB, UB)	p-value
**Model 1**	-0.436 (-2.858. +1.985)	0.723
**Model 2**	-0.660 (-3.067, +1.747)	0.590
**Model 3**	-0.432 (-2.831, +1.967)	0.724
**Model 4**	-0.402 (-2.736, +1.931)	0.735
**Model 5**	-0.957 (-3.232, +1.319)	0.409
**Model 6**	-1.006 (-3.282, +1.270)	0.386
*** Statistically significant p-value ≤ 0.05**		
**Model 1: GDS, Dysphoria tertile. Model 2: +Age. Model 3: +Apathy score. Model 4: +ACE score. Model 5: +General health. Model 6: +Physically active days per week**		

**Table S6: Linear Regression of GDS Score and Subjective Cognitive Changes**		
**Independent Variable**	CCI	
**Model**	Beta Coefficient (95% CI LB, UB)	p-value
**Model 1**	+6.164 (+5.056, +7.271)	≤0.001 *
**Model 2**	+6.222 (+5.125, +7.319)	≤0.001 *
**Model 3**	+5.718 (+4.596, +6.841)	≤0.001 *
**Model 4**	+5.403 (+4.205, +6.601)	≤0.001 *
**Model 5**	+5.361 (+4.159, +6.563)	≤0.001 *
*** Statistically significant p-value ≤ 0.05**		
**Model 1: GDS Tertile. Model 2: +Age. Model 3: +ACE score. Model 4: +General health. Model 5: +Physically active days per week**		

Table S7: Linear Regression of Dysphoria and Subjective Cognitive Changes		
**Independent Variable**	CCI	
**Model**	Beta Coefficient (95% CI LB, UB)	p-value
**Model 1**	+0.836 (-0.521, +2.192)	0.227
**Model 2**	+0.955 (-0.394, +2.304)	0.165
**Model 3**	+0.478 (-0.748, +1.703)	0.445
**Model 4**	+0.467 (-0.744, +1.678)	0.449
**Model 5**	+0.690 (-0.505, +1.884)	0.258
**Model 6**	+0.702 (-0.494, +1.899)	0.249

*** Statistically significant p-value ≤ 0.05**
Model 1: GDS, Dysphoria Tertile. Model 2: +Age. Model 3: + Apathy Score. Model 4: +ACE score. Model 5: +General health. Model 6: +Physically active days per week
